# Genome-Wide Transcriptome Profiles of Rice Hybrids and Their Parents

**DOI:** 10.3390/ijms151120833

**Published:** 2014-11-13

**Authors:** Zhiguo E, Shanshan Huang, Yuping Zhang, Lei Ge, Lei Wang

**Affiliations:** 1 China National Rice Research Institute, Hangzhou 310006, China; E-Mails: ezhiguo@caas.cn (Z.E); zhangyuping02@caas.cn (Y.Z.); 2 ReaderBio Company, Ltd., Beijing 100193, China; E-Mail: shanshanhuang@readerbio.com; 3 Nantong Agricultural College, Nantong 226007, China; E-Mail: gelei0711@gmail.com

**Keywords:** transcriptome profile, hybrid rice, allele-specific expressed genes, differentially-expressed genes

## Abstract

Heterosis is a widely studied phenomenon in several plant species. However, its genetic basis still remains to be elucidated. In this study, we used RNA-seq data from two rice genotypes and their reciprocal hybrids, and used a combination of transcriptome profiling and allele-specific expression analysis to identify genes that are differentially expressed in the hybrids and their parents or expressed in an allele-specific manner. The differentially expressed genes (DEGs) were identified by a pairwise comparison of the four genotypes. Detailed annotation of DEGs suggested that these genes showed enrichment in some gene ontology categories, and they tend to have tissue-specific expression patterns compared to all genes. A total of 1033 (10.24%) of 10,195 genes with informative single nucleotide polymorphism (SNPs) were identified as ASE genes. These allele-specific expessed (ASE) genes showed a broader expression breadth suggesting that they function in diverse developmental stages. Among 1033 ASE genes, we also identified 45 ASE transcription factors belonging to 17 transcription factor families. These ASE transcription factors may act *in trans* to regulate gene expression in filial 1 (F_1_) hybrids. Our analyses provide a comprehensive transcriptome profile of rice hybrids and their parents, and would be a useful resource for the rice research community.

## 1. Introduction

Heterosis or hybrid vigor is the improved or superior biological function of any hybrid progeny in comparison to their homozygous parents. Superior phenotypic performance can be observed in diverse agronomical traits, such as biomass and grain yield.

Although heterosis has been largely exploited in modern breeding programs, such as in rice and maize, the molecular mechanisms underlying heterosis remain very elusive. The transcriptome diversity of the super-hybrid rice varieties (*LYP9*, *Liangyou2186*, and *Xieyou9308*) and their parents was examined. Different tissues at various stages of development were analyzed by whole-genome oligonucleotide microarray [1], serial analysis of gene expression (SAGE) [2] and RNA-seq technology [3]. Some studies focused on the epigenetic control of reciprocal filial 1 (F_1_) hybrid of Nipponbare (NPB) and 93-11 [4,5]; however, these two studies did not provide a detailed transcriptome annotation.

Phenotypic variation between hybrids and their parents could be a result of variation in the complex gene expression network. In rice, thousands of genes were differentially expressed between the hybrid and its parents [3]. In alfalfa hybrids, the majority of the genes with a non-additive pattern of expression fell outside the parental range [6]. Detailed detection and annotation of the transcriptome of reciprocal hybrid and their parents are important for understanding the mechanisms underlying hybrid vigor. Additionally, the breadth of expression of genes that are differentially expressed in parents and their hybrid progenies remain to be elucidated. This is important because if these genes showed more tissue- or developmental stage-specific expression patterns, these tissues or developmental stages could be explored in future studies.

Complementation contributes to transcriptome complexity in maize (*Zea mays* L.) hybrids relative to their inbred parents [7]. Genome-wide analysis of allele-specific expressed (ASE) genes is as available as the widely-used RNA-seq. The knowledge of allelic contributions to gene expression in the hybrid helps us understand hybrid vigor. ASE genes have been studied at different developmental stages on a global transcriptomic scale in rice [8]. For example, in rice, both strongly inherited ASE genes and the differentially expressed genes between hybrids and parents were identified [4]. Furthermore, allelic expression bias in hybrids was correlated with parental differences [5]. However, the breadth of expression of ASE genes was largely unknown.

Rice, one of the most important food crops, is an excellent model to study heterosis. Here, using the public RNA-seq data from two rice genotypes and their reciprocal F_1_ hybrids [4], we give a genome-wide transcriptome profile of rice hybrids and their parents. Thousands of genes were identified as differentially expressed between the F_1_ hybrids and parents. Using RNA-seq data from different developing stages, we could estimate the tissue expression patterns of genes. Parental effect was also observed. ASE genes, especially ASE transcriptome factors were identified in our study. The transcriptome profile generated by our study would help us understand the variations in gene expression between the F_1_ hybrids and their parents and give better insight into the mechanism of heterosis.

## 2. Results and Discussion

### 2.1. Results

#### 2.1.1. Alignment of RNA-seq Data

RNA-seq data provides transcriptome profiles of rice hybrids and their parents. RNA-seq data for seedlings of NPB, 93-11 and their reciprocal hybrids have been generated in previous studies [4]. A total of 311 million reads were generated for 4 fully expanded leaves from 6-week-old plants of the four genotypes. We downloaded reads of four genotypes from Sequence Read Archive (SRA) in National Center for Biotechnology Information (NCBI). Reads from RNA-seq data were aligned to the NPB reference genome (Build 6.1). About 248 (79.74%) million reads were uniquely aligned to Nipponbare reference genome, ranging from 55 to 68 million reads for the four genotypes (Table 1). We also found that 24,017 of 35,119 (68.38%) annotated genes in the NPB reference genome were detected by at least one uniquely mapped read in at least one of the four genotypes, with 33,505 annotated genes in NPB, 32,531 in 93-11, 34,895 in NPB × 93-11 and 93-11 × NPB (Figure 1). In both hybrids, NPB × 93-11 and 93-11 × NPB, more genes were detected with RNA-seq reads than their parents. A similar result (Figure S1) was obtained when we used RNA-seq data of seedling shoots at the four-leaf stage from 4-week-old plants of the four genotypes [5]. These results indicate that more genes were expressed in hybrid progenies than in their parents.

**Table 1:** Statistics of alignment results for RNA-seq data.

Samples	# of Reads	Length of Reads	# of Bases	# of Uniquely Mapped Reads	Percentage of Uniquely Mapped Reads
NPB	77,019,774	50	3,850,988,700	55,413,403	71.95%
9311	74,384,043	50	3,719,202,150	58,116,517	78.13%
NPB × 93-11	81,735,674	50	4,086,783,700	68,473,811	83.77%
93-11 × NPB	78,026,146	50	3,901,307,300	66,122,696	84.74%

# means the number.

**Figure 1:**
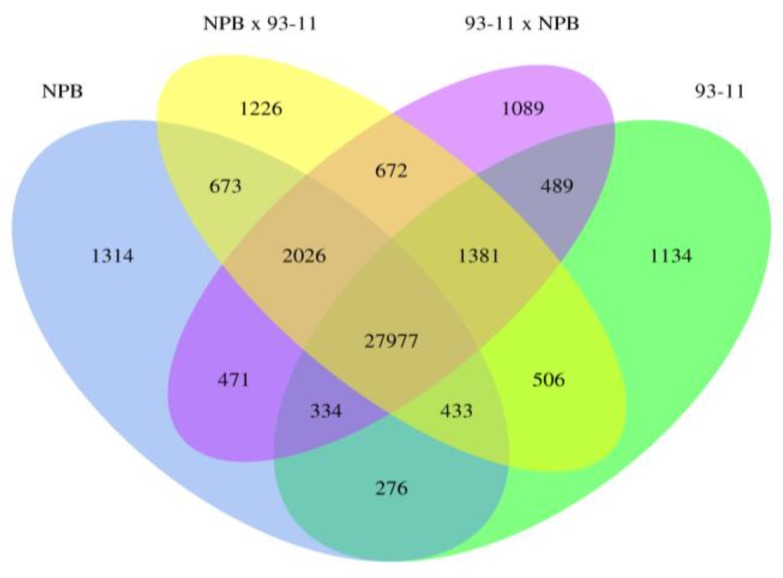
The number of genes that were detected by at least one read is indicated for each genotype in the Venn diagram. Four genotypes are shown in different colors. # means the number.

#### 2.1.2. Global Analysis of Gene Expression

**Figure 2:**
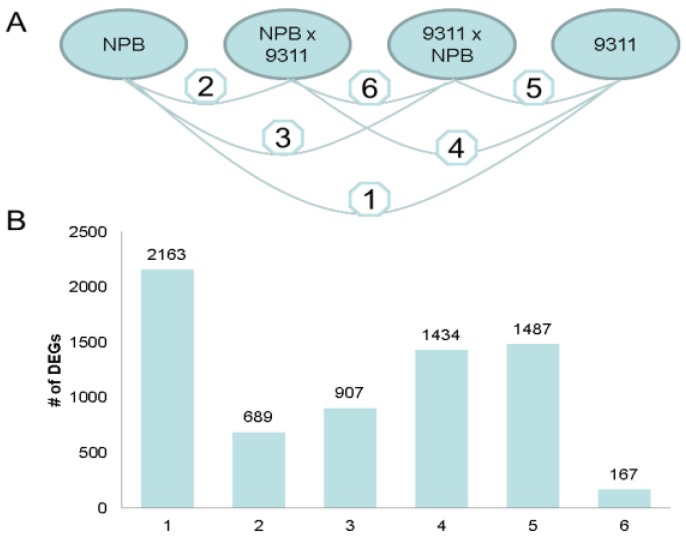
Identification of differentially expressed genes in all six pairwise comparisons between four genotypes. (**A**) All possible pairwise comparisons between the four genotypes; (**B**) Number of differentially expressed genes (DEGs) in all possible pairwise comparison.

To examine the diversity of the transcriptome of the four genotypes, the differentially expressed genes (DEGs) were identified for all possible pairwise comparisons of the four genotypes [9] (See Materials and Methods; Figure 2A). When controlling the false discovery rate (FDR) at 5%, 2163 DEGs between two parents NPB and 93-11 were identified, which was much higher than the number (167) of DEGs identified between the two hybrids, NPB × 93-11 and 93-11 × NPB (Figure 2B). The number of DEGs by comparison between parents and hybrids ranged from 689 to 1487. Comparisons between the two parents and their hybrids showed fewer DEGs than comparison between two parents, but more than the comparison of the reciprocal hybrids (Figure 2B).

Majority of genes that were identified as differentially expressed between inbred and hybrids were also identified as differentially expressed between the two parents. There were 531 DEGs identified both in the comparison between NPB and NPB × 93-11, and the comparison between NPB and 93-11 × NPB (Figure S2), of which 90% were also identified as DEGs between two parents (Figure S2). Similar results were also observed in the comparison between 93-11 and NPB × 93-11, and the comparison between 93-11 and 93-11 × NPB (Figure S2).

#### 2.1.3. Parental Influence on Hybrids in Rice

Imprinted genes in plants were mainly identified in endosperms [10]. We observed that the number of DEGs identified between NPB × 93-11 and its mother NPB was lower than that of the DEGs identified between 93-11 × NPB and its father NPB. Similar results were observed when comparing two hybrids to 93-11 (Figure 2B). Our results together with previous reports strongly suggest a differential parental influence on the progeny transcriptomes. Similar results in rice and maize indicate that parental influence on the hybrids is a conserved phenomenon [7].

Since hybrids had conserved parental influence, it would be interesting to identify imprinted genes in the seedlings. However, we did not find any imprinted genes in the rice seedlings. Therefore, despite the parental influence on the hybrids, the parental influence on the seedlings differs from the parental influence on the endosperm. Furthermore, the maternal influence was higher than the paternal influence. Thus, the parental influence on the hybrid seedlings may not be gene-specific but genotype-specific.

#### 2.1.4. Functional Annotation of Differentially Expressed Genes (DEGs) by Pairwise Comparisons

**Figure 3:**
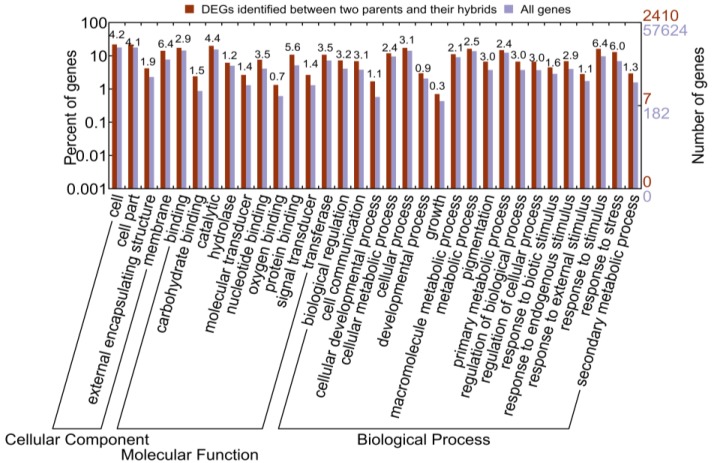
The distribution of gene ontology (GO) terms assigned to DEGs between parent and hybrid. All genes were compared. The p-value is below the significant level of 0.05. Percentage of enrichment is also shown in the figure.

DEGs between the parent and hybrid should play an important role in hybrid vigor. To investigate the characteristics of these DEGs, we first performed gene ontology (GO) classification of the DEGs. We found that these genes could be classified into a diversity of GO categories, such as cell, cell part and organelle in the cellular component category, binding, catalytic, and molecular transducer in the molecular function category, cellular process, metabolic process and response to stimulus in the biological process category (Supplemental Figure S3). We also compared the GO categories for the DEGs between parents and hybrids *versus* all genes. Using WEGO (Web Gene Ontology Annotation Plot) [11], we observed that the DEGs between parents and hybrids are significantly over-represented in special GO categories, such as biological regulation, response to stimulus and response to stress in biological process category (Figure 3). Genes located in the over-represented categories may contribute to the hybrid vigor.

**Figure 4:**
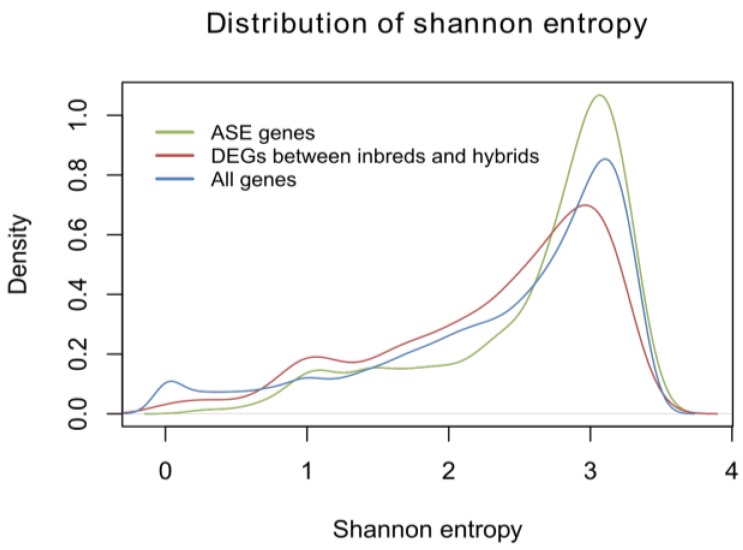
The distribution of Shannon entropy for DEGs between inbreds and hybrids. All genes were detected.

Shannon entropy was used as an index to measure expression breadth [12]. High and low Shannon entropies refer to broad and tissue-specific patterns, respectively (see Materials and Methods). The RNA-seq data from 11 tissues (Table S1) was used to investigate the expression breadth of DEGs between inbreds and hybrids. FPKM (Fragments per Kilobase of Transcripts per Million Mapped Reads) were calculated for each gene in all 11 tissues. We further investigated the tissue expression patterns of these DEGs across different tissues. DEGs exhibit significantly lower Shannon entropy than all other genes (*p* value < 2.2 × 10^−16^, Student’s *t*-Test; Figure 4), suggesting that DEGs tend to have tissue-specific expression patterns.

Hybrid vigor was thought to be involved in many genes and many different pathways. Genes with expression levels that are highly correlated are biologically interesting because they imply common regulatory mechanisms or participation in similar biological processes. Since we wished to identify putative pathways related to hybrid vigor, we used RNA-seq data from 11 tissues to construct a co-expression network (see Materials and Methods), based on weighted correlation network analysis [13]. Using this analysis, we could describe the correlation patterns among genes and finding clusters (modules) of highly correlated genes across the 11 samples [13]. We obtained 19 co-expression modules with gene numbers ranging from 80 to 7183. We observed that DEGs between parent and hybrid existed in all 19 modules, but were not equally distributed. Modules 1, 2, 7, 11, 12 and 44 were under-represented by DEGs between the parent and hybrid. On the other hand, the modules 3, 4, 10 and 36 were over-represented by DEGs between the parent and hybrid (Figure S4).

**Figure 5:**
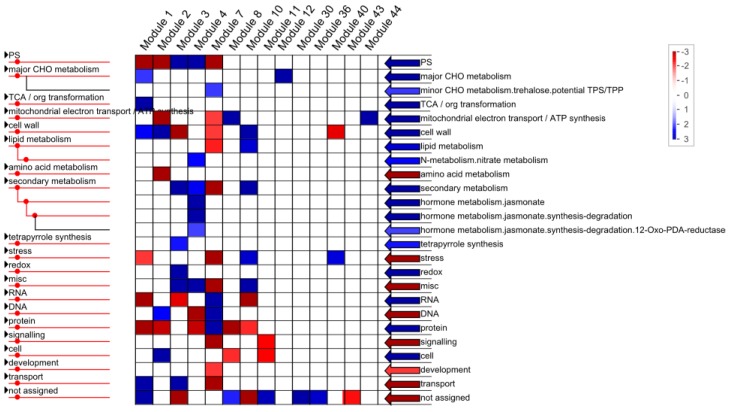
GO classifications of different co-expression modules using PageMan. Red color indicates over-represented, whereas blue color indicates down-represented.

Functional enrichment analysis with regard to known gene ontologies in MapMan were used to understand the biological significance of the module genes and to identify putative pathways related to hybrid vigor [14]. Some GO terms were over-represented in special modules, such as GO terms named major carbohydrate (CHO) metabolism, tricarboxylic acid (TCA) transformation, nitrate metabolism, tetrapyrrole synthesis and redox (Figure 5). Although the GO term, named major carbohydrate (CHO), metabolism was over-represented when all genes were considered, it was only over-represented in module 1and module 12. The GO term named TCA/org transformation, which was otherwise over-represented when all genes were considered, was only over-represented in module 1.

Some GO terms, which are otherwise over-represented when all genes are considered, were over-represented in special modules, but under-represented in other modules (Figure 5). For example, for the GO terms named photosystem, mitochondrial ATP synthesis coupled electron transport (An increased activity or efficiency of the F_1_Fo–ATP synthase could contribute to the phenomenon known as hybrid vigor in the F_1_ hybrid [15], cell wall, lipid metabolism and secondary metabolism, *etc.*). The GO term PS was also over-represented in module 3 and module 4, but was underrepresented in module 1, 2 and 7. Similarly, the GO term named cell wall, was only over-represented in module 1, module 2 and module 8 but under-represented in module 40.

#### 2.1.5. Identification of Allele-Specific Expressed Genes

The complementation model is one of the major models that explain hybrid vigor. The allele specific expression pattern is a special instance of complementation. RNA-seq data from the reciprocal hybrids of NPB and 93-11 help us to obtain a high-quality set of ASE genes.

We assigned informative reads to parental alleles in the two hybrids (see Materials and methods) to achieve insight into allelic expression patterns in rice hybrids. Only uniquely mapped reads were used, and 46,986 (7.77%) of 604,729 single nucleotide polymorphisms (SNPs) were analyzed with sufficient parental reads. Overall, 33,217 exonic SNPs in 10,195 gene were analyzed (see Materials and methods). The ratio of maternal reads for SNPs analyzed exhibited expected allelic value, and its distribution fitted a normal distribution, indicating a majority of genes were bi-allelic expressed (Figure S5).

Allele-specific expressed genes were identified on the basis of the following criteria: (1) the ratio of allelic reads from the two parents exceeding the expected ratio (1:1) in both hybrids; (2) percentage of either allelic reads should be higher than 80% (see Materials and Methods). Under these criteria, 1033 (10.24%) of 10,195 genes were identified as ASE genes.

**Figure 6:**
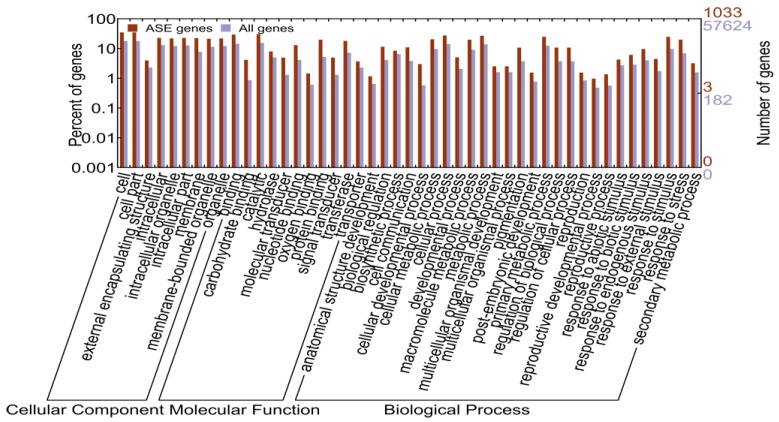
The distribution of GO terms assigned to allele-specific expressed (ASE) genes in hybrids. All genes were compared.

To investigate whether these ASE genes were over- or under-represented in special biological processes, we performed gene ontology enrichment analysis using WEGO (Figure 6). With all genes as background, we observed that ASE genes showed GO enrichment in diverse GO subcategories (Figure 6). ASE genes showed significant enrichment in the terms of cell part, membrane-bounded organelle and intracellular organelle in cell component category (Figure 6). In the molecular function categories, ASE genes showed a significant enrichment in terms of catalytic activity, transporter activity, binding, and molecular transducer activity (Figure 6). In the biological process category, the ASE genes showed a significant enrichment in terms of biological regulation, response to stimulus and developmental process (Figure 6).

We further investigated the tissue expression patterns of these ASE genes across different tissues. Shannon entropy was used as an index to measure expression breadth [13]. ASE genes exhibited significantly higher Shannon entropy than all genes (*p* value < 2.2 × 10^−16^; Figure 4), suggesting that ASE genes tend to have a broader expression breadth.

#### 2.1.6. Allele-Specific Expressed Transcription Factors

Transcription factors bind to *cis*-regulatory DNA sequences of target genes to regulate the binding of RNA polymerase. Interaction of transcription factors and variable *cis*-regulatory elements in the promoter may be a good molecular model to explain heterosis [16]. In *Arabidopsis*, the transcription factors such as MYB-like transcription factors (Late Elongated Hypocotyl (LHY) and Circadian Clock Associated (CCA1)) that are involved in the circadian rhythm are associated with heterosis. ASE transcription factors may play an important role in heterosis. Intriguingly, we observed that the ASE genes enriched in the GO term named nucleotide binding in molecular function subcategory. DNA binding domain is a major feature for a transcription factor. To determine whether ASE transcription factors exist, transcription factors were identified across the rice genome [17]. In rice, 2172 transcription factors belonging to 57 transcription factor families were identified, with the basic helix-loop-helix (bHLH) family being the largest.

Of the 2127 transcription factors, 400 were associated with informative SNPs and 45 of 2172 transcription factors showed allelic expression patterns. The 45 ASE transcription factors belonged to 17 transcription factor family, with 15 of the 45 transcription factors belonging to the Far-Red Impaired Response 1 (FAR1) transcription factor family (Figure S6). These ASE transcription factors may function as *trans*-factors to regulate gene expression in F_1_ hybrids.

### 2.2. Discussion

Complementation contributes to transcriptome complexity in maize (*Zea mays* L.) hybrids relative to their inbred parents [7]. Because of complementation, more genes are expressed in hybrids than their parents. We observed that more genes were detected in reciprocal hybrids as compared to their parents in rice. Single parent expression, a special instance of the complementation model, can be observed in the reciprocal hybrid in maize. More than 800 genes showed single parent expression pattern, most of which are thought to be a consequence of differentially expressed genes between two parents, rather than the presence or absence of variations. Hybrid plants have phenotypic advantages because of the expression of a larger number of genes in hybrids than their parents.

In maize, previous results suggest that the maternal influence exceeds the paternal influence, independent of their genotypes [7]. Previous studies have reported that hybrid gene expression patterns resemble the transcriptome of maternal [18] or paternal [19] genes in maize hybrids. Unlike parental influence on endosperm in plants, we did not find imprinting-like expression patterns in rice seedlings. This result corroborates previous studies that the imprinting pattern of genes exclusively happens in the endosperm.

Allelic bias of gene expression has been reported in previous studies in F_1_ hybrids [5,7]. ASE genes were enriched in GO terms named cell part, membrane-bounded organelle and intracellular organelle. Cell part, membrane-bounded organelle and intracellular organelles were important throughout the developmental stages. Heterosis can change or get modified over time or during growth and development of plants and animals [16]. Heterosis characteristics in vegetative tissues such as vigorous growth in seedlings and roots may not be directly correlated with higher seed yields because different sets of genes in the biological pathways control vegetative growth and reproductive development, although some pathways are intricately related [20]. So DEGs between parent and hybrid showed tissue-specific expression patterns and may contribute more to seedling than other tissues such as reproductive tissues (seed size and 50-seed weight, *etc.*), which indicates that DEGs between parents and hybrids should be studied in diverse developmental stages, rather than just one stage. Meanwhile, our results showed that ASE genes have a broader expression breadth as compared to DEGs, suggesting that ASE genes function in diverse developmental stages. These results resemble their functional GO annotations.

Transcription factors increased or repressed the expression of their target genes by binding to specific *cis*-regulatory elements. Extensive sequence variations, especially insertions and deletions (INDELs) can result in the formation/disruption of *cis*-regulatory elements in rice [16]. Combination of differentially expressed transcription factors and variations in *cis*-regulatory elements may bring distinct modes of gene expression patterns in the F_1_ hybrid progeny [16]. Although a conservative estimation, we identified 45 ASE transcription factors. As with variations of *cis*-regulatory elements, the differential expression of the alleles of transcription factors could influence gene expression patterns *in trans* and contribute to different modes of gene action in F_1_ hybrids [16].

## 3. Materials and Methods

### 3.1. RNA-seq Data

*Oryza sativa ssp. japonica* (NPB), *O. sativa ssp. indica* (93-11), and their reciprocal crosses (NPB × 93-11 and 93-11 × NPB) were used in this study. RNA-seq data for the four genotypes (NPB, 93-11, NPB × 93-11 and 93-11 × NPB) were downloaded from Sequence Read Archive (SRA) with accession SRP013556 [4]. The tissues used for RNA-seq were fully expanded leaves from 6-week-old plants [4]. The reciprocal hybrids (NPB × 93-11 and 93-11 × NPB) used show significant growth vigor compared with their parents [5].

We also investigated the tissue expression patterns of genes in rice, for which RNA-seq data from 11 tissues were downloaded from Sequence Read Archive (SRA) with accession SRP008821 (Table S1).

### 3.2. Alignment of RNA-seq Data

SRA format was converted into fastq format using the SRA tool kit. Bowtie [21] was used to build the index for rice reference genome. Reads from the fastq format files of RNA-seq data for the four genotypes were mapped to the *Oryza sativa ssp. japonica* (cv. Nipponbare) version 6.1 reference genome [22] using TopHat [23].

### 3.3. Identification of Differentially Expressed Genes

After alignment of the RNA-seq data, only uniquely mapped reads were retained for subsequent analysis. The count data, presented as a table which reports the number of reads that have been assigned to a gene for each genotype, was generated by multiBamCov in BEDtools software [24]. Differentially expressed genes were identified by DESeq based on the negative binomial distribution, with variance and mean linked by local regression [9]. A gene was identified as differentially expressed if the adjusted *p*-value was below 0.05 and there was at least a twofold change in each possible pairwise comparison.

### 3.4. Identification of Allele-Specific Expressed Genes and Imprinted Genes

SNP between NPB and 93-11 were sincerely provided by Matteo Pellegrini (Los Angeles, CA, USA) [4]. Simulated 93-11 reference genome was constructed using SNPs between NPB and 93-11. Reads from hybrid transcriptomes were aligned with the NPB and 93-11 reference genome, respectively using Tophat with parameter: --bowtie1 -g 1. Allelic read depths were obtained. ASE-pattern SNPs were defined as: (1) either allele had a significant bias in both hybrids (*p* value < 0.05, Fisher’s exact test); (2) more than 80% of reads should come from the allele of NPB genome (or 93-11 genome) in both hybrids. ASE genes were defined as genes with ASE-pattern SNPs. SNPs showing imprinting status were defined as: (1) either allele had a significant bias in both hybrids (*p* value < 0.05, Fisher’s exact test); (2) more than 80% of reads should come from maternal (or paternal) alleles in both hybrids. Imprinted genes were defined as genes with SNPs showing imprinting status.

### 3.5. Calculation of Fragments per Kilobase of Transcripts per Million Mapped Reads (FPKM)

To calculate FPKM, cufflinks was used [25] based on the bam format files generated by TopHat.

### 3.6. Construction of Co-Expression Network

WGCNA (Weighted correlation network analysis) package provides R functions for weighted correlation network analysis, e.g., co-expression network analysis of gene expression data [13]. Correlation networks are increasingly being used in biology to analyze large, high-dimensional data sets [13]. FPKM was log*2* transformed. For a gene, FPKM in at least one tissue should be greater than 1.

### 3.7. Mapman

MapMan “Bins” were assigned to protein-coding genes from version 6.1 reference genome by the Mercator web application. PageMan analysis was performed to annotate genes in the modules of the co-expression network.

### 3.8. Calculation of Shannon Entropy

Shannon entropy was used as an index to measure the expression breadth [12]. High and low Shannon entropy refers to broad and tissue-specific patterns, respectively.

### 3.9. Gene Ontology Classification

GO term of genes from rice version 6.1 reference genome were downloaded. GO classifications for a set of genes were performed by the Web Gene Ontology Annotation Plot (WEGO) [11]. All genes in the rice genome were used as background for significance testing. The file with GO terms for rice genes were downloaded from web sites (ftp://ftp.plantbiology.msu.edu/pub/data/Eukaryotic_Projects/o_sativa/annotation_dbs/pseudomolecules/version_6.1/all.dir/all.GOSlim_assignment.gz).

### 3.10. Identification of Transcription Factors Using Profile hidden Markov models (HMMER3)

HMMER 3.0 software was used to identify the rice transcription factors [26], which were classified into 58 families in PlantTFDB [27]. Custom perl scripts were used to extract the gene names of the identified transcription factors from the HMMER output file.

## 4. Conclusions

Our results provide a detailed transcriptome profile and annotation of rice hybrids and their parents. DEGs between inbred and hybrid rice may vary among different developmental stages because of their lower Shannon entropy, whereas ASE genes exhibit stable expression patterns across different developmental stages and may play more extensive important roles than DEGs between inbred and hybrid rice. Mechanisms that cause DEGs between inbred and hybrid and ASE genes in F_1_ hybrid need more detailed investigation.

## Supplementary Materials

Supplementary materials can be found at: http://www.mdpi.com/1422-0067/15/11/20833/s1.

## References

[1] Wei G., Tao Y., Liu G., Chen C., Luo R., Xia H., Gan Q., Zeng H., Lu Z., Han Y. (2009). A transcriptomic analysis of superhybrid rice *LYP9* and its parents. Proc. Natl. Acad. Sci. USA.

[2] Song G.-S., Zhai H.-L., Peng Y.-G., Zhang L., Wei G., Chen X.-Y., Xiao Y.-G., Wang L., Chen Y.-J., Wu B. (2010). Comparative transcriptional profiling and preliminary study on heterosis mechanism of super-hybrid rice. Mol. Plant.

[3] Zhai R., Feng Y., Wang H., Zhan X., Shen X., Wu W., Zhang Y., Chen D., Dai G., Yang Z. (2013). Transcriptome analysis of rice root heterosis by RNA-seq. BMC Genomics.

[4] Chodavarapu R.K., Feng S., Ding B., Simon S.A., Lopez D., Jia Y., Wang G.-L., Meyers B.C., Jacobsen S.E., Pellegrini M. (2012). Transcriptome and methylome interactions in rice hybrids. Proc. Natl. Acad. Sci. USA.

[5] He G., Zhu X., Elling A.A., Chen L., Wang X., Guo L., Liang M., He H., Zhang H., Chen F. (2010). Global epigenetic and transcriptional trends among two rice subspecies and their reciprocal hybrids. Plant Cell.

[6] Scotti C., Carelli M., Calderini O., Panara F., Gaudenzi P., Biazzi E., May S., Graham N., Paolocci F., Arcioni S. (2011). Agronomic and molecular analysis of heterosis in alfalfa. Plant Genet. Resour..

[7] Paschold A., Jia Y., Marcon C., Lund S., Larson N.B., Yeh C.-T., Ossowski S., Lanz C., Nettleton D., Schnable P.S. (2012). Complementation contributes to transcriptome complexity in maize (*Zea mays* L.) hybrids relative to their inbred parents. Genome Res..

[8] Zhai R., Feng Y., Zhan X., Shen X., Wu W., Yu P., Zhang Y., Chen D., Wang H., Lin Z. (2013). Identification of transcriptome SNPs for assessing allele-specific gene expression in a super-hybrid rice Xieyou9308. PLoS One.

[9] Anders S., Huber W. (2010). Differential expression analysis for sequence count data. Genome Biol..

[10] Luo M., Taylor J.M., Spriggs A., Zhang H., Wu X., Russell S., Singh M., Koltunow A. (2011). A genome-wide survey of imprinted genes in rice seeds reveals imprinting primarily occurs in the endosperm. PLoS Genet..

[11] Ye J., Fang L., Zheng H., Zhang Y., Chen J., Zhang Z., Wang J., Li S., Li R., Bolund L. (2006). WEGO: A web tool for plotting GO annotations. Nucleic Acids Res..

[12] Schug J., Schuller W.P., Kappen C., Salbaum J.M., Bucan M., Stoeckert C.J. (2005). Promoter features related to tissue specificity as measured by Shannon entropy. Genome Biol..

[13] Langfelder P., Horvath S. (2008). WGCNA: An R package for weighted correlation network analysis. BMC Bioinform..

[14] Thimm O., Blasing O., Gibon Y., Nagel A., Meyer S., Kruger P., Selbig J., Muller L.A., Rhee S.Y., Stitt M. (2004). MAPMAN: A user-driven tool to display genomics data sets onto diagrams of metabolic pathways and other biological processes. Plant J..

[15] Sabar M., Gagliardi D., Balk J., Leaver C.J. (2003). ORFB is a subunit of F1F(O)-ATP synthase: Insight into the basis of cytoplasmic male sterility in sunflower. EMBO Rep..

[16] Zhang H.-Y., He H., Chen L.-B., Li L., Liang M.-Z., Wang X.-F., Liu X.-G., He G.-M., Chen R.-S., Ma L.-G. (2008). A genome-wide transcription analysis reveals a close correlation of promoter INDEL polymorphism and heterotic gene expression in rice hybrids. Mol. Plant.

[17] Ni Z., Kim E.-D., Ha M., Lackey E., Liu J., Zhang Y., Sun Q., Chen Z.J. (2009). Altered circadian rhythms regulate growth vigour in hybrids and allopolyploids. Nature.

[18] Guo M., Rupe M.A., Danilevskaya O.N., Yang X., Hu Z. (2003). Genome-wide mRNA profiling reveals heterochronic allelic variation and a new imprinted gene in hybrid maize endosperm. Plant J..

[19] Swanson-Wagner R.A., DeCook R., Jia Y., Bancroft T., Ji T., Zhao X., Nettleton D., Schnable P.S. (2009). Paternal dominance of *trans*-eQTL influences gene expression patterns in maize hybrids. Science.

[20] Chen Z.J. (2010). Molecular mechanisms of polyploidy and hybrid vigor. Trends Plant Sci..

[21] Langmead B., Trapnell C., Pop M., Salzberg S.L. (2009). Ultrafast and memory-efficient alignment of short DNA sequences to the human genome. Genome Biol..

[22] Ouyang S., Zhu W., Hamilton J., Lin H., Campbell M., Childs K., Thibaud-Nissen F., Malek R.L., Lee Y., Zheng L. (2007). The TIGR rice genome annotation resource: Improvements and new features. Nucleic Acids Res..

[23] Trapnell C., Pachter L., Salzberg S.L. (2009). TopHat: Discovering splice junctions with RNA-seq. Bioinformatics.

[24] Quinlan A.R., Hall I.M. (2010). BEDTools: A flexible suite of utilities for comparing genomic features. Bioinformatics.

[25] Trapnell C., Hendrickson D.G., Sauvageau M., Goff L., Rinn J.L., Pachter L. (2013). Differential analysis of gene regulation at transcript resolution with RNA-seq. Nat. Biotechnol..

[26] Eddy S.R. (1998). Profile hidden Markov models. Bioinformatics.

[27] Zhang H., Jin J., Tang L., Zhao Y., Gu X., Gao G., Luo J. (2011). PlantTFDB 2.0: Update and improvement of the comprehensive plant transcription factor database. Nucleic Acids Res..

[28] Davidson R.M., Gowda M., Moghe G., Lin H., Vaillancourt B., Shiu S.-H., Jiang N., Buell C.R. (2012). Comparative transcriptomics of three Poaceae species reveals patterns of gene expression evolution. Plant J..

